# A Comparative Study on Phytochemical Fingerprint of Two Diverse *Phaseolus vulgaris* *var.* Tondino del Tavo and Cannellino Bio Extracts

**DOI:** 10.3390/antiox11081474

**Published:** 2022-07-28

**Authors:** Azzurra Stefanucci, Giuseppe Scioli, Lorenza Marinaccio, Gokhan Zengin, Marcello Locatelli, Angela Tartaglia, Alice Della Valle, Angelo Cichelli, Ettore Novellino, Stefano Pieretti, Adriano Mollica

**Affiliations:** 1 Department of Pharmacy, University of Chieti–Pescara “G. d’Annunzio”, Via dei Vestini 31, 66100 Chieti, Italy; giuseppe.scioli@unich.it (G.S.); lorenza.marinaccio@unich.it (L.M.); marcello.locatelli@unich.it (M.L.); angela.tartaglia@unich.it (A.T.); alice.dellavalle@unich.it (A.D.V.); adriano.mollica@unich.it (A.M.); 2 Department of Biology, Science Faculty, Selcuk University, 42250 Konya, Turkey; gokhanzengin@selcuk.edu.tr; 3 Department of Innovative Technologies in Medicine and Dentistry, University of Chieti–Pescara “G. d’Annunzio”, Via dei Vestini 31, 66100 Chieti, Italy; angelo.cichelli@unich.it; 4 Department of Medicine and Surgery, Università Cattolica del Sacro Cuore, 00168 Rome, Italy; ettore.novellino@unicatt.it; 5 NGN Healthcare-New Generation Nutraceuticals s.r.l., Torrette Via Nazionale 207, 83013 Mercogliano, Italy; 6 National Centre for Drug Research and Evaluation, Istituto Superiore di Sanità, 00161 Rome, Italy; stefano.pieretti@iss.it

**Keywords:** extraction, fractions, phenols, enzymes, HPLC profile, protein, flavonoids

## Abstract

Common bean (*Phaseolus vulgaris*) represents one of the most famous foods with antiobesity activity showing a significant efficacy against fat accumulation, insulin resistance and dyslipidaemia. In this work, two Italian varieties of common bean, i.e., Tondino del Tavo and Cannellino Bio, from the centre of Italy were studied to characterise their phenolic profile by HPLC-PDA in relation to different fractions after a straightforward extraction procedure. Antioxidant property and enzymatic inhibition power were also evaluated in order to delineate a possible biological profile. Results show a considerable phenolic content (0.79 and 1.1 µg/mg of 3-hydroxybenzoic acid for hexane extract of Tondino del Tavo and Cannellino Bio, respectively; 0.30 µg/mg *p*-coumaric acid for *n*-hexane extract of Tondino del Tavo) for both varieties, and a strong antioxidant activity according to the major phenolic concentration of the extracts. The anti-inflammatory activity of the decoction extracts was also investigated through a zymosan-induced edema formation assay, revealing a moderate ability for both of them. These preliminary data prompt us to further explore the nutrient components of these two varieties in the future.

## 1. Introduction

**Figure 1:**
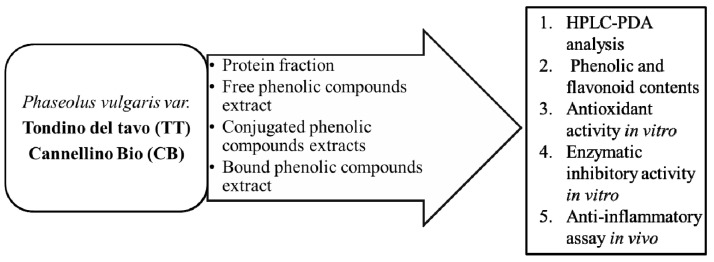
Workflow of comparative analysis for diverse extracts of *Phaseolus vulgaris var*: TT, Tondino del Tavo; CB, Cannellino Bio.

Metabolic syndrome is a pathological condition of obese people characterised by hypercholesterolemia, hepatic steatosis, abdominal obesity and many concomitant biochemical alterations such as the accumulation of cytoplasmatic triglycerides, insulin resistance and changes in glucose metabolism [1,2]. A new strategy for the management of metabolic syndrome consists of the use of nutraceuticals [3]; in particular, common bean extracts (*Phaseolus vulgaris*) exhibit some beneficial effects against metabolic syndrome [1,2]. The common bean is the most diffuse legume in the world and represents half of the total legume consumption in the human diet [4]. It is rich in proteins, fibres, unsaturated fatty acids, vitamins, minerals and flavonoids [4,5]. Its extracts exhibit the capacity to inhibit α-amylase, thus reducing body absorption of carbohydrates, leading to a decrease in body weight, glycemia, triglycerides and cholesterol blood level [1,2,4,5]. The antiobesity activity of common bean extracts is confirmed by many different preclinical studies in rat models. Tormo and coworkers isolated and purified an inhibitor of human α-amylase from common bean extracts, demonstrating the hypoglycaemic and anorexigenic power of this inhibitor in vivo [6]. Neil and coworkers reported that the consumption of common bean extracts plays a role in body weight control by reducing abdominal fat accumulation in mice models, with an increase in intestinal mass, without modification of crypts height and mucin content [7]. No differences in weight loss between the group treated with common bean extract in the slimming regimen (dietary modification, exercise) and the control group (slimming regimen without common bean extracts) during a short period of time were registered in this study [8]. A meta-analysis of the antiobesity activity of common bean extracts confirmed the statistically significant effect of weight loss in humans treated with common bean extracts. In fact, *Phaseolus vulgaris* extracts reduced body weight by 1.08 kg and body fat by 3.26 kg (both results had a 95% CI) [9]. The antiobesity activity of common bean extract is due to the presence of an α-amylase inhibitor isoform I called *phaseolamin* (α-AI), which is able to block α-amylase digestive enzyme activity, preventing carbohydrate metabolism and absorption [2,4,8,10]. De Gouveia and coworkers tested commercial *phaseolamin in vitro* and in vivo, demonstrating its capacity to inhibit α-amylase activity in vitro, thus reducing blood glucose levels [11]. SDS-PAGE analysis showed the presence of many types of proteins such as α-AI (16–11 kDa) and *phaseolin* (50–35 kDa), the most representative glycoprotein in common bean, and *phytohemagglutinin* (35–25 kDa); no toxicity was observed in rats, while low hemagglutination activity was detected on all types of human erythrocytes [11]. In addition, Barret and Udani confirmed the role of *phaseolamin* in inhibiting α-amylase and in reducing body weight and fat accumulation in humans [12,13]. Other examples of beans with antidiabetic activity are mung and adzuki beans. Mung beans contain a series of polyphenols exhibiting α-amylase and α-glucosidase inhibition activity, suggesting a potential control of postprandial glucose levels [14]. Adzuki bean extracts contain polyphenols, which are able to reduce total hepatic lipids and triglycerides accumulation [15]. Polyphenols can reduce the hydrolysis of *phaseolin*, thus interfering with its digestion [16]. In this work, we focused our attention on two varieties of *Phaseolus vulgaris*, i.e., Tondino del Tavo and Cannellino Bio, from the centre of Italy. The Tavo is an Abruzzo river that originates from the Apennine peak of the Gran Sasso; it flows in the hilly area among lush valleys. The round bean from Tavo is small and round like a pea, with a candid pearly colour ranging from milky white to ivory; it has excellent nutritional properties and a very thin skin, which allows for faster cooking and easy digestion [17]. Irrigation is essential in times of low rainfall, which coincides with the period of pod enlargement. The beans are kept in jute bags, away from sources of light and heat. Cannellino Bio from Colfiorito (Perugia, Umbria, Italy) is very smooth; it is characterised by an ovoid shape and uniform snow-white colour. It is considered one of the best varieties for production and organoleptic qualities. It is part of the culinary and cultural heritage of the Umbria region. This legume can be considered an excellent substitute for meat and pasta; although it contains very little fat, it is highly caloric, representing an important source of carbohydrates and proteins, and due to the presence of fibre, helps to maintain normal cholesterol levels [18]. In this preliminary work, we performed a separation of diverse fractions through a well-established extraction procedure for each variety (Figure 1).

Each fraction was analysed through HPLC-PDA, showing a significant phenolic content (0.79 and 1.1 µg/mg of 3-hydroxybenzoic acid for *n*-hexane extract of Tondino del Tavo and Cannellino Bio, respectively; 0.30 µg/mg *p*-coumaric acid for *n*-hexane extract of Tondino del Tavo) in both varieties. Antioxidant assays revealed a strong activity according to their major phenolic concentration. Then, decoction extracts were tested for their anti-inflammatory activity through zymosan assay, indicating a moderate ability to reduce oedema formation.

## 2. Materials and Methods

### 2.1. Materials

Tondino del Tavo (Loreto Aprutino, Pescara, Abruzzo, Italy) and Cannellino Bio (Colfiorito, Perugia, Umbria, Italy) were selected as the common bean variety (*Phaseolus vulgaris*) belonging to the genus *Phaseolus.* They were collected and stored according to their use for human consumption. Tondino del Tavo was purchased at the Passeri Carlo Company (Loreto Aprutino, Pescara) and Cannellino Bio (Colfiorito, Perugia) at a local market. Methanol, ethanol, *n*-hexane, ethyl acetate and acetonitrile for HPLC, NaOH, HCl and NaCl were purchased at Sigma Aldrich (Milan, Italy). Gallic acid, catechin, chlorogenic acid, *p*-hydroxybenzoic acid, vanillic acid, epicatechin, syringic acid, *p*-coumaric acid, rutin, ferulic acid, sinapinic acid, 3-hydroxybenzoic acid, 3-hydroxy-4-methoxybenzoc acid, naringin, 2,3-dimethoxybenzoic acid, *o*-coumaric acid, quercetin, cinnamic acid, carvacrol and naringenin were purchased at Sigma Aldrich (Milan, Italy). The centrifuge was an Eppendorf 5702 (Hamburg, Germany). The lyophilisator was a Buchi L-100 (BUCHI Italia s.r.l. Cornaredo, Italy).

### 2.2. Sample Preparation

One hundred grams of common beans for each variety were lyophilised, and the obtained material was weighted, shredded into a mortar, transferred into a plastic vial far from sunlight and stored at 20 °C. Around one gram of Cannellino Bio and Tondino del Tavo was powdered with a blender and freeze-dried. Five hundred milligrams of Cannellino Bio and Tondino del Tavo was suspended in 70% ethanol/water solution (20 mL) and boiled for 5 min. The powder was filtered and freeze-dried again. The prepared extracts were obtained with 18% and 20% yields, respectively.

### 2.3. Extraction Method

#### 2.3.1. Protein Fraction

Five grams of lyophilised material was placed into a reaction flask with 40 mL of NaCl 1.5% m/v solution (8 mL/g of sample) and stirred for 4 h at room temperature [17]. The supernatant was transferred into five vials of 6 mL each and then centrifugated for 90 min at 4.400 rpm. After centrifugation, the supernatant was transferred into a reaction flask, acidified at pH 6 and heated at 70 °C for 20 min under reflux. Then, the supernatant was placed into eight vials of 6 mL each and centrifuged for 40 min at 4.400 rpm. The supernatant was separated by the precipitate, and it was diluted with ethanol in a ratio of 3:7 and then put into a fridge at 4 °C for 60 min. Finally, the supernatant was centrifuged again for 15 min at 4.400 rpm. The supernatant was wasted, and the solid precipitated was dried under a high vacuum overnight [17]. The yield of the white bean protein extract was 2.61 g/100 g and 2.57 g/100 g of dried bean for Tondino del Tavo (TT) and Cannellino Bio (CB), respectively.

#### 2.3.2. Free Phenolic Compounds Fraction

Free phenols extraction was performed following the method developed by Telles et al., in which 5 grams of lyophilised material was placed into a reaction flask with 17 mL of methanol and stirred for 1 h [18]. The mixture was rested for 15 min, after which the solvent was removed, 17 mL of methanol was added to the powder and the mixture was stirred again for 90 min. This procedure was repeated twice. The tree solutions of methanol were gathered, transferred into four vials of 6 mL each and centrifuged for 5 min at 4.400 rpm. The supernatant was placed into four vials of 6 mL each, and then a solution of ZnSO_4_ 0.1M (2.1 mL) and Ba(OH)_2_ solution 0.1M (2.1 mL) were added for clarification. The mixtures obtained were rested for 30 min and then centrifuged for 5 min at 4.400 rpm. This procedure was repeated twice. After clarification, the supernatants were placed together into a reaction flask, and the solvent was removed by rotavapor. The yield was 1.36 g/100 g and 1.12 g/100 g of dried bean for Tondino del Tavo (TT) and Cannellino Bio (CB), respectively.

#### 2.3.3. Conjugated Phenolic Compounds Fraction

Ten grams of lyophilised material was weighed, transferred into a reaction flask with 33.3 mL of ethanol (10 mL/3 g lyophilised material) and stirred for 10 min. The solid was filtered on a Buckner funnel, extracted with 33.3 mL of ethanol and stirred for 10 min. The supernatant was centrifuged for 10 min at 4.400 rpm in seven vials of 6 mL each. Finally, the supernatant was placed into a reaction flask, and the solvent was evaporated with a rotavapor. The yield was 2.14 g/100 g and 2.08 g/100 g of dried bean for Tondino del Tavo (TT) and Cannellino Bio (CB), respectively.

#### 2.3.4. Bound Phenolic Compounds Fraction

Half of the conjugated phenolic compounds fraction (Section 2.3.3) was added to 16.6 mL of *n*-hexane (10 mL/3 g of product) and stirred in a reaction flask for 10 min. The obtained supernatant was placed into four vials and centrifuged for 35 min at 4.400 rpm. The supernatant was wasted, and the precipitate was dried in a high vacuum. The solid was transferred into a reaction flask with 70 mL of NaOH 4M and stirred for 3 h. Then, the solution was acidified at pH 1.0 with HCl 6M, centrifuged and placed into a separation funnel. The solution was extracted two times with ethyl acetate, placed into a flask and dried with dry sodium sulphate for 15 min. The solvent was removed in a rotavapor until a white solid was obtained. The yield of the white bean extract was 1.16 g/100 g and 1.05 g/100 g of dried bean for Tondino del Tavo (TT) and Cannellino Bio (CB), respectively.

### 2.4. HPLC-PDA Analysis

The HPLC-PDA procedure was performed on a model 600 solvent pump coupled with a 2996 PDA detector (Waters Spa, Milford, MA, USA) [19]. A C18 reversed-phase column (Prodigy ODS-3, 4.6 × 150 mm, 5 µm; Phenomenex, Torrance, CA, USA), thermostated at 30 ± 1 °C using a Jetstream2 Plus column oven, was used for the separation. The UV/Vis acquisition wavelength was set in the range of 200–500 nm. The quantitative analyses were achieved at a maximum wavelength for each compound, in particular 271 nm, 278 nm, 324 nm, 256 nm, 260 nm, 278 nm, 274 nm, 275 nm, 278 nm, 310 nm, 256 nm, 324 nm, 315 nm, 285 nm, 299 nm, 275 nm, 276 nm, 367 nm, 280 nm, 276 nm, 290 nm and 275 nm for gallic acid, catechin, chlorogenic acid, 4-hydroxybenzoic acid, vanillic acid, epicatechin, syringic acid, 3-hydroxybenzoic acid, 3-hydroxy-4-methoxybenzaldehyde, *p*-coumaric acid, rutin, sinapinic acid, *t*-ferulic acid, naringin, 2,3-dimethoxybenzoic acid, benzoic acid, *o*-coumaric acid, quercetin, harpagoside, *t*-cinnamic acid, naringenin and carvacrol, respectively. The injection volume was 20 μL. The mobile phases were directly *online* degassed by using Biotech DEGASi, mod. Compact (LabService, Anzola dell’Emilia, Italy). The mobile phases consisted of solution A (3% solution of acetic acid in water) and solution B (3% solution of acetic acid in acetonitrile) at a ratio of 93:7 (*v*:*v*), and the gradient mode was applied (93–2% eluent A in 45 min). The flow rate was set at 1 mL/min throughout the analysis. Empower v.2 software (Waters Spa, Milford, MA, USA) was used to collect and analyse the raw data obtained after the sample suspension (accurately weighed) in the mobile phase, sonicated, centrifuged at 12,000 rpm and directly injected (20 µL) into the HPLC-PDA system.

### 2.5. Total Phenolic and Flavonoid Content

The total concentrations of phenols and flavonoids were determined using previously described methods [20]. Total phenolic content was expressed as mg gallic acid equivalents (GAE)/g dry extract, while total flavonoids were expressed as mg rutin equivalents (RE)/g dry extract. The assays were performed in triplicate, and ANOVA (Tukey’s test) was used to determine the differences in the extracts.

### 2.6. Antioxidant and Enzyme Inhibitory Assays

The antioxidant activity of the extracts was determined using a variety of assays: 1,1-diphenyl-2-picrylhydrazyl (DPPH) and 2,2′-azino-bis(3-ethylbenzothiazoline)-6-sulfonic acid (ABTS) radical scavenging capacity (CUPRAC), ferric ion reducing antioxidant power (FRAP), metal chelating ability (MCA) and phosphomolybdenum assay (PBD) [21]. The data for the DPPH, ABTS, CUPRAC and FRAP assays were expressed in mg Trolox equivalents (TE)/g extract, whereas the data for MCA and PDA were expressed in mg EDTA equivalents (EDTAE)/g extract and mmol TE/g extract, respectively. In the cholinesterase assays, galantamine was used as a positive control, and data were expressed as mg galantamine equivalents (GALAE)/g extract. In the tyrosinase inhibitory assay, kojic acid was used as a standard inhibitor, and the results were expressed as mg kojic acid equivalents (KAE)/g extract [22]. In the antidiabetic assays, acarbose was chosen as an inhibitor of both amylase and glucosidase, and the results are expressed as mmol acarbose equivalents (ACAE)/g extract. AChE and BChE are acetylcholinesterase and butyrylcholinesterase enzymes, respectively.

### 2.7. Data Analysis

The assays were performed in triplicate, and ANOVA (Tukey’s test) was used to determine the differences in the extracts. The relationship between biological activity assays and total bioactive components was demonstrated using Pearson correlation analysis. The analysis was carried out utilising Graph Pad Prism (version 9.2, GraphPad Software 2365 Northside Dr. Suite 560 San Diego, CA 92108, USA). For the in vivo assay, statistical analysis was performed by using two-way ANOVA followed by Dunnett’s multiple comparisons test.

### 2.8. Zymosan-Induced Oedema Formation

Male CD-1 mice (Harlan, Italy) of 3–4 weeks (25 g) were used for all the experiments. Mice were housed in colony cages under standard conditions of light, temperature and relative humidity for at least 1 week before starting experimental sessions. All experiments were performed according to Legislative Decree 27/92 and approved by the local ethics committee (approval number 198/2013-B). Mice received a subcutaneous (s.c.) administration (20 μL/paw) of zymosan A (2.5% *w/v* in saline) into the dorsal surface of the right hind paw. Paw volume was measured three times before the injections and at 1, 2, 3, 4 and 24 h thereafter using a hydroplethysmometer apparatus (UgoBasile, Via Giuseppe di Vittorio, 2, 21036 Gemonio VA Italy). The increase in paw volume was then evaluated as the percentage difference between the paw volume at each time point and the basal paw volume [23]. Cannellino Bio and Tondino del Tavo decoction extracts were dissolved in DMSO:saline (ratio 1:3 *v/v*) and were administered s.c. into the dorsal surface of the right hind paw at a dose of 100 μg/20 μL paw 15 min before or 150 min after zymosan.

## 3. Results and Discussion

To evaluate the phenolic content of the different *Phaseolus vulgaris* extracts, HPLC-PDA analysis was used, and a small number of phenolic substances was found in each type of extract, as reported in Table 1. The major phenolic content of these secondary metabolites was found in the *n*-hexane extract of Tondino del Tavo (#4TT), in which 3-hydroxybenzoic acid (0.79 µg/mL) and *p*-coumaric acid (0.30 µg/mg) were detected. The other metabolites were found in low concentrations (below the limit of quantification). Similar results were obtained for Cannellino Bio, in which the major phenolic content was also measured in #4CB, in which 3-hydroxybenzoic (1.1 µg/mg) was the most abundant (#4CB). Quantities below the quantification limit of *p*-OH benzoic acid, sinapinic acid and *t*-ferulic acid were found in #4TT (for HPLC traces see Supplementary Material). The quantities of *p*-coumaric acid and sinapinic acid in #2CB/TT and #3CB/TT were also found to be below the quantification limits. The low concentration of *t*-ferulic acid and sinapinic acid was also reported in other studies based on a comparative analysis among diverse Phaseolus species [24].

Diverse factors influence the TPC, TFC and composition of common beans, for example, the environmental conditions, genotype, storage and processing methods. Several works stressed the major influence of genotype over location in the determination of the TPC of common beans [25]. Compared with TPC, there are fewer reports about the effect of germination on the TFC of common beans; Xue et al. reported that the TFC of black beans increased after a prolonged germination time, reaching the highest value on the fifth day [26]. Bioactive compounds such as gallic aldehyde, protocatechuic acid, protocatechuic aldehyde, *p*-hydroxybenzoic acid, *p*-hydroxybenzoic aldehyde, *trans*-feruloyl aldaric acid and sinapoyl aldaric acid were found in the insoluble fractions of *Phaseolus vulgaris* L., while no hydroxybenzoic compounds were identified in the insoluble fraction of the germinated beans [27]. Furthermore, extraction techniques have a key role in the recovery of polyphenols. In this context, several matrices have been used, such as raw beans, seed coats, cotyledons and germinated and fermented common beans. Indeed, there is a lack of data about the optimal extraction condition of common bean polyphenols. On the other hand, the extraction solvent is crucial to reaching high-efficiency polyphenol extraction. Methanol/water/acid systems are most commonly used for phenolic acids; however, these are not suitable for gaining a soluble phenolic fraction [28]. In order to obtain polyphenol-rich extracts, they can be purified to remove impurities by diverse types of adsorbents [29]. The Folin–Ciocalteu test did not show a significant total phenolic content in both varieties of *Phaseolus vulgaris* according to HPLC analysis. The major total phenolic content was found in #4CB (25.42 mg of GAE/g) and #4TT (11.59 mg GAE/g) (Table 2).

In vitro antioxidant activity was evaluated with DPPH and ABTS assays and reducing power with CUPRAC and FRAP tests. The best antioxidant activity was found in #4CB (55.96 mg TE/g in ABTS), which is active on DPPH (6.65 mg TE/g) as well as #4TT (ABTS: 16.11 mg TE/g). These extracts reported the best reductive activity in CUPRAC (64.69 mg TE/g for CB and 37.05 mg TE/g for TT). Similar results were obtained in FRAP (#4CB: 42.94 mg TE/g; #4TT: 21.01 mg TE/g) (Table 3).

These results are directly linked to the major phenolic concentration obtained through the extraction procedure applied (Section 2.3). In the present work, the total phenolic and flavonoid contents in the tested extracts were examined by colourimetric methods. The highest level of total phenols was determined in the bound phenolic extract of Cannellino Bio (#4CB: 25.42 mg GAE/g), followed by the conjugated phenolic extract of Cannellino Bio (#3CB: 11.84 mg GAE/g) and bound phenolic extract of Tondino del Tavo (#4TT: 11.59 mg GAE/g). However, #2TT contained the highest level of total flavonoids (1.68 mg RE/g). Generally, the tested extracts exhibited low levels of flavonoids (<1 mg RE/g); however, different levels of total phenols and flavonoids in *Phaseolus vulgaris* samples have been observed in the literature [30,31,32,33]. In a recent paper by Rossi et al., the effects of germinating time and gastrointestinal digestion on the chemical composition and biological activities of two common *Phaseolus vulgaris* cultivars were analysed, revealing a strong correlation between their phenolic contents and soil composition [34]. Looking at the radical scavenging and reducing power data, the bound extracts of both varieties were the most active; this could be explained by the higher total phenolic content with respect to the other fractions.

In the phosphomolybdenum assay, the activity of analysed samples can be ranked as follows: #4CB (1.55 mmol TE/g) > #4TT (1.18 mmol TE/g) > #3TT (1.13 mmol TE/g) > #3CB (1.10 mmol TE/g) > #2CB (0.59 mmol TE/g) > #2TT (0.56 mmol TE/g) > #1TT (0.26 mmol TE/g) > #1CB (0.12 mmol TE/g). This behaviour was almost the same for CUPRAC and FRAP assays. Otherwise, in the other antioxidant assays, the best metal chelating abilities were recorded for #1CB (16.51 mg EDTAE/g) and #1TT (16.24 mg EDTAE/g). Several researchers have reported metal chelating abilities for some peptides and the antioxidant properties of *Phaseolus vulgaris* extracts [35,36]. Furthermore, Hernandez-Guerrero et al. studied the correlation between secondary metabolites identified through the NMR technique and the biological activities of *Phaseolus vulgaris* cultivars [37]. Generally, coloured bean cultivars exhibited superior antioxidant activity compared with white common beans [38]. The extracts were also tested for their inhibitory activity against cholinesterases, amylase, glucosidase and tyrosinase (Table 4).

**Figure 2:**
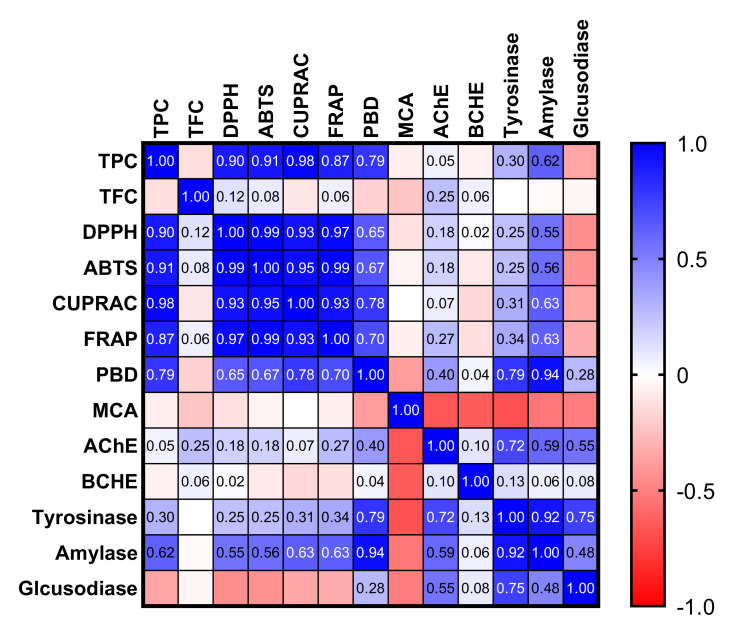
Pearson correlation values between biological activity assays (*p* < 0.05): TPC, total phenolic content; TFC, total flavonoid content; ABTS, 2,2′-azino-bis(3-ethylbenzothiazoline)-6-sulphonic acid; DPPH, 1,1-diphenyl-2-picrylhydrazyl; CUPRAC, cupric reducing antioxidant capacity; FRAP, ferric reducing antioxidant power; AChE, acetylcholinesterase; BChE, butyrylcholinesterase; MCA, metal chelating ability; PBD, phosphomolybdenum.

In AChE inhibition, the most active extracts were #3CB and #2TT, while #3TT and #1TT were not active on AChE. Regarding BChE inhibitory assay, the best action was observed in #1TT (4.30 mg GALAE/g). Tyrosinase is a key enzyme in the synthesis of melanin; it plays a vital role in the progression of hyperpigmentation problems [39]. All extracts exhibited inhibitory effects on tyrosinase, and the most active one was #4TT (66.44 mg KAE/g). Amylase and glucosidase are considered antidiabetic enzymes, and their inhibition is an important mechanism for controlling blood glucose in diabetic patients [40]. In amylase inhibition, the bound phenolic extracts displayed the strongest inhibitory effects (#4CB: 0.57 mmol ACAE/g; #4TT: 0.43 mmol ACAE/g), and #1CB and #4CB were not active on glucosidase. These data are partially in line with the recent literature devoted to the study of *Phaseolus vulgaris* extracts. Schisano et al. reported the amylase inhibitory effects of the Controne ecotype of *Phaseolus vulgaris,* for which the inhibition was found to be 70% [41]. Furthermore, Micheli et al. investigated the amylase inhibition ability of *Phaseolus vulgaris* in mice, highlighting its significant activity in alleviating diabetic symptoms [2]. Fonseca-Hernández et al. investigated tyrosinase inhibitory effects of black bean polyphenol extracts. The purified extracts (IC50: 0.143–0.147 mg/mL) displayed stronger inhibitory activity than crude extracts (IC50: 2.59–9.92 mg/mL) [42]. In order to complete our panel of biological activity screening, in vivo zymosan-induced oedema formation was applied for decoction extracts of Cannellino Bio and Tondino del Tavo, with the aim of highlighting possible anti-inflammatory activity (Figure 2). Cannellino Bio and Tondino del Tavo reduced oedema formation when administered 15 min before or 150 min after zymosan, but these effects did not reach statistical significance.

**Figure 3:**
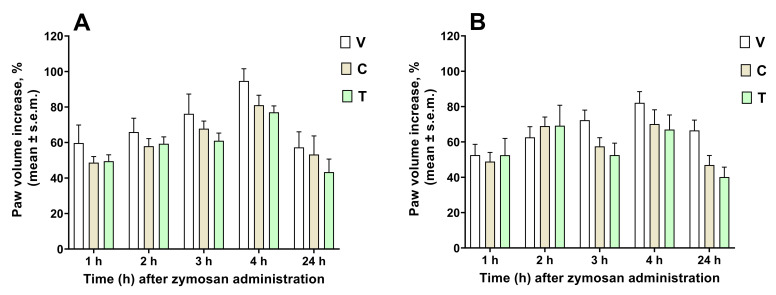
Effects induced by Cannellino Bio (C) and Tondino del Tavo (T) decoction extracts administered s.c. into the mice hind paw at the dose of 100 μg/20 μL paw, 15 min before (**A**) or 150 min after (**B**) zymosan (2.5% *w*/*v* in saline, 20 μL/paw) administration in the same paw. Statistical analysis was performed by using two-way ANOVA followed by Dunnett’s multiple comparisons test. C and T induced a reduction in zymosan-induced oedema, but this effect did not reach statistical significance both in (**A**,**B**). *N* = 7.

In order to obtain new insights between the total bioactive components and the antioxidant and enzyme-inhibiting effects of the tested extracts, we performed a correlation analysis. The results are shown in Figure 3. In fact, the antioxidant properties correlate strongly with the total phenolic levels of the tested extracts, with the exception of metal chelating ability. In particular, the correlation values for the free radical scavenger and reducing power assays were higher than 0.8. The results clearly showed that the phenolic compounds were the main contributors to the antioxidant properties of the tested extracts. Consistent with our findings, many researchers have reported that phenolics in plant extracts are the key players in antioxidant properties [43,44]. The conflicting results in terms of metal chelating ability can be explained by the presence of nonphenolic chelating agents such as polysaccharides or peptides [35]. However, the correlation values of enzyme inhibition assays differed from antioxidant assays. Among the assays, only the amylase inhibition assay showed a moderate correlation with total phenol content (*R* > 0.6). Other enzymes were almost weakly linked with total phenolic content. At this point, the observed ability to inhibit enzymes could be related to nonphenolic enzyme inhibitors such as alkaloids or terpenoids [44,45]. However, we suggest that the individual components could be isolated through further analysis techniques, then their enzyme inhibitory effects could be better evaluated in future studies.

Findings by Cardador-Martinez et al. demonstrated the potent antimutagenic activity and antioxidant properties of diverse fractions of *Phaseolus vulgaris* obtained using different extraction solvents, further supporting our hypothesis [46]. Nonetheless, our study demonstrates the feasibility of producing fractions of bean with considerable levels of phenolic antioxidants by means of this methodology, which could be potentially applied to the extraction of protein-rich legumes, such as soybeans, lentils and split peas and Graminacee-derived natural products such as barley and grain. Overall, these results could be partially explained by the extraction process applied; changing the solvents used to treat the lyophilised samples could represent a useful strategy to improve the extraction yield and, at the same time, to obtain fractions rich in bioactive compounds such as flavonoids.

## 4. Conclusions

In this short communication, an easy and straightforward extraction procedure was assessed and applied to the separation of different fractions of two Italian varieties of *Phaseolus vulgaris*, i.e., Tondino del Tavo and Cannellino, which were analysed by quantitative HPLC-PDA to determine their phenolic fingerprint. Results show considerable total phenolic content for both varieties and growing antioxidant activity related to the major phenolic content of each extract. On the other hand, a low enzymatic inhibition profile against AChE, BChE and glucosidase enzymes was also detected for all of them, with the exception of the activity found against tyrosinase enzyme. Despite these preliminary data, we are confident in further development of this work starting from the enrolled extraction procedure, which can be employed to determine the protein content and complete phenolic profile for each of them in the future.

## Figures and Tables

**Table 1:** Quantitative analysis (μg/mg) of the *Phaseolus Vulgaris* extracts.

Sample ID	*p*-OH Benzoic Acid	3-Hydroxybenzoic Acid	*p*-Coumaric Acid	Sinapinic Acid	*t*-Ferulic Acid
#1TT					
#2TT			BLQ	BLQ	
#3TT			BLQ	BLQ	
#4TT	BLQ	0.79 ± 0.08	0.30 ± 0.02	BLQ	BLQ
#1CB					
#2CB				BLQ	
#3CB					
#4CB	BLQ	1.1 ± 0.1	BLQ	BLQ	BLQ

Values are reported as media ± standard deviation of three parallel experiments: **BLQ**, below limit of quantification; **TT**, Tondino del Tavo; **CB**, Cannellino Bio.

**Table 2:** Phenolic content of the tested samples.

Samples	TPC (mg GAE/g)	TFC (mg RE/g)	DPPH (mg TE/g)	ABTS (mg TE/g)
#1TT	6.63 ± 0.11 ^c^	0.02 ± 0.01 ^e^	n.a.	0.76 ± 0.03 ^e^
#2TT	3.46 ± 0.06 ^d^	1.68 ± 0.01 ^a^	n.a.	n.a.
#3TT	10.86 ± 0.11 ^b^	0.06 ± 0.02 ^cde^	n.a.	0.71 ± 0.01 ^e^
#4TT	11.59 ± 0.31 ^b^	0.07 ± 0.02 ^cd^	1.04 ± 0.02 ^b^	16.11 ± 0.07 ^b^
#1CB	7.22 ± 0.02 ^c^	0.61 ± 0.02 ^b^	n.a.	2.90 ± 0.22 ^c^
#2CB	3.32 ± 0.02 ^d^	0.08 ± 0.01 ^c^	n.a.	n.a.
#3CB	11.84 ± 0.03 ^b^	0.02 ± 0.01 ^de^	n.a.	1.30 ± 0.02 ^d^
#4CB	25.42 ± 1.23 ^a^	0.61 ± 0.03 ^b^	6.65 ± 0.11 ^a^	55.96 ± 0.01 ^a^

Values are reported as media ± DS of three parallel experiments: TPC, total phenolic content; TFC, total flavonoid content; GAE, gallic acid equivalent; RE, rutin equivalent; TE, Trolox equivalent; n.a., not active. Different superscripts (a–e) in same column indicate significant differences in the samples (by ANOVA (Tukey’s test), *p* < 0.05).

**Table 3:** Antioxidant activity of the tested samples.

Samples	CUPRAC (mg TE/g)	FRAP (mg TE/g)	Chelating Activity (mg EDTAE/g)	PBD (mg TE/g)
#1TT	19.97 ± 0.57 ^f^	6.17 ± 0.07 ^e^	16.24 ± 0.07 ^a^	0.26 ± 0.02 ^d^
#2TT	15.14 ± 0.13 ^g^	7.40 ± 0.28 ^d^	3.34 ± 0.87 ^cd^	0.56 ± 0.09 ^c^
#3TT	30.43 ± 0.13 ^c^	6.96 ± 0.03 ^de^	7.64 ± 0.21 ^b^	1.13 ± 0.05 ^b^
#4TT	37.05 ± 0.92 ^b^	21.01 ± 0.71 ^b^	15.68 ± 2.31 ^a^	1.18 ± 0.05 ^b^
#1CB	24.14 ± 1.53 ^e^	6.78 ± 0.02 ^de^	16.51 ± 0.06 ^a^	0.12 ± 0.01 ^e^
#2CB	16.95 ± 0.67 ^g^	9.48 ± 0.69 ^c^	2.65 ± 0.52 ^d^	0.59 ± 0.03 ^c^
#3CB	27.48 ± 0.35 ^d^	6.83 ± 0.12 ^de^	1.81 ± 0.10 ^d^	1.10 ± 0.02 ^b^
#4CB	64.69 ± 0.55 ^a^	42.94 ± 0.03 ^a^	5.63 ± 0.10 ^bc^	1.55 ± 0.01 ^a^

Values are reported as media ± DS of three parallel experiments: TE, Trolox equivalent; EDTAE, EDTA equivalent. Different superscripts (a–g) in same column indicate significant differences in the samples (by ANOVA (Tukey’s test), *p* < 0.05).

**Table 4:** Enzymatic inhibition of the tested samples.

Samples	AChE (mg GALAE/g)	BChE (mg GALAE/g)	Tyrosinase (mg KAE/g)	Amylase (mg TE/g)	Glucosidase (mmol ACAE/g)
#1TT	0.02 ± 0.01 ^d^	4.30 ± 0.01 ^a^	15.07 ± 0.57 ^d^	0.05 ± 0.01 ^e^	n.a.
#2TT	2.57 ± 0.03 ^a^	3.96 ± 0.18 ^bc^	57.94 ± 0.23 ^c^	0.30 ± 0.02 ^d^	1.11 ± 0.04 ^a^
#3TT	n.a.	3.85 ± 0.01 ^c^	62.02 ± 0.20 ^b^	0.39 ± 0.02 ^bc^	1.15 ± 0.01 ^a^
#4TT	2.18 ± 0.03 ^b^	n.a.	66.44 ± 0.14 ^a^	0.43 ± 0.01 ^b^	1.15 ± 0.01 ^a^
#1CB	n.a.	0.61 ± 0.07 ^e^	15.73 ± 1.15 ^d^	0.05 ± 0.00 ^e^	n.a.
#2CB	2.51 ± 0.07 ^a^	4.07 ± 0.08 ^b^	60.93 ± 0.40 ^b^	0.30 ± 0.01 ^d^	1.14 ± 0.02 ^a^
#3CB	2.57 ± 0.04 ^a^	3.76 ± 0.04 ^cd^	65.45 ± 0.49 ^a^	0.38 ± 0.02 ^c^	1.11 ± 0.01 ^a^
#4CB	1.90 ± 0.02 ^c^	3.57 ± 0.01 ^d^	61.36 ± 0.67 ^b^	0.57 ± 0.03 ^a^	n.a.

Values are reported as media ± DS of three parallel experiments: GALAE, galantamine equivalent; KAE, kojic acid equivalent; ACAE, acarbose equivalent; AChE, acetylcholinesterase; BChE: butyrylcholinesterase; n.a., not active. Different superscripts (a–e) in same column indicate significant differences in the samples (by ANOVA (Tukey’s test), *p* < 0.05)

## Data Availability

Data is contained within the article and supplementary material.

## Supplementary Materials

The following supporting information can be downloaded at: https://www.mdpi.com/article/10.3390/antiox11081474/s1.
