# Supplementary material for: A Comparative Study on Phytochemical Fingerprint of Two Diverse Phaseolus vulgaris var. Tondino del Tavo and Cannellino Bio Extracts
Source: Antioxidants (Basel). 2022 Jul 28;11(8):1474. doi: 10.3390/antiox11081474 (PMC9405002; doi:10.3390/antiox11081474)
Supplement: Supplementary file 1 [file antioxidants-11-01474-s001.zip › antioxidants-1828602-supplementary.pdf]

**SUPPORTING INFO**

# A comparative study on phytochemical fingerprint of two di-verse *Phaseolus vulgaris* var. Tondino del Tavo and Cannellino Bio extracts

Azzurra Stefanucci[1,*], Giuseppe Scioli[1], Lorenza Marinaccio[1], Gokhan Zengin[2], Marcello Locatelli[1], Angela Tartaglia[1], Alice Della Valle[1], Angelo Cichelli[3], Ettore Novellino[4,5], Stefano Pieretti[6], Adriano Mollica[1]

[1]Department of Pharmacy, University of Chieti–Pescara “G. d’Annunzio”, Via dei Vestini 31, Chieti 66100, Italy; a.stefanucci@unich.it; giuseppe.scioli@unich.it; lorenza.marinaccio@unich.it; marcello.locatelli@unich.it; angela.tartaglia@unich.it; alice.dellavalle@unich.it; adriano.mollica@unich.it

[2]Selcuk University, Science Faculty, Department of Biology, Campus, 42250 Konya, Turkey; gokhan-zengin@selcuk.edu.tr

[3]Department of Innovative Technologies in Medicine and Dentistry, University of Chieti–Pescara “G. d’Annunzio”, Via dei Vestini 31, Chieti 66100, Italy; angelo.cichelli@unich.it

[4]Department of Medicine and Surgery, Università Cattolica del Sacro Cuore, 00168 Rome, Italy; ettore.novellino@unicatt.it

[5]NGN Healthcare-New Generation Nutraceuticals s.r.l., Torrette Via Nazionale 207, 83013 Mercogliano, Italy;

[6]National Centre for Drug Research and Evaluation, Istituto Superiore di Sanità, 00161 Rome, Italy; stefano.pieretti@iss.it

*Correspondence: a.stefanucci@unich.it

# Tondino del Tavo
# 4TT Extract

| p-OH benz | 3 OH benz | p-coumaric acid | Sinapinic acid | t-ferulic acid |
|---|---|---|---|---|
| 256 | 295 | 309 | 324 | 315 |

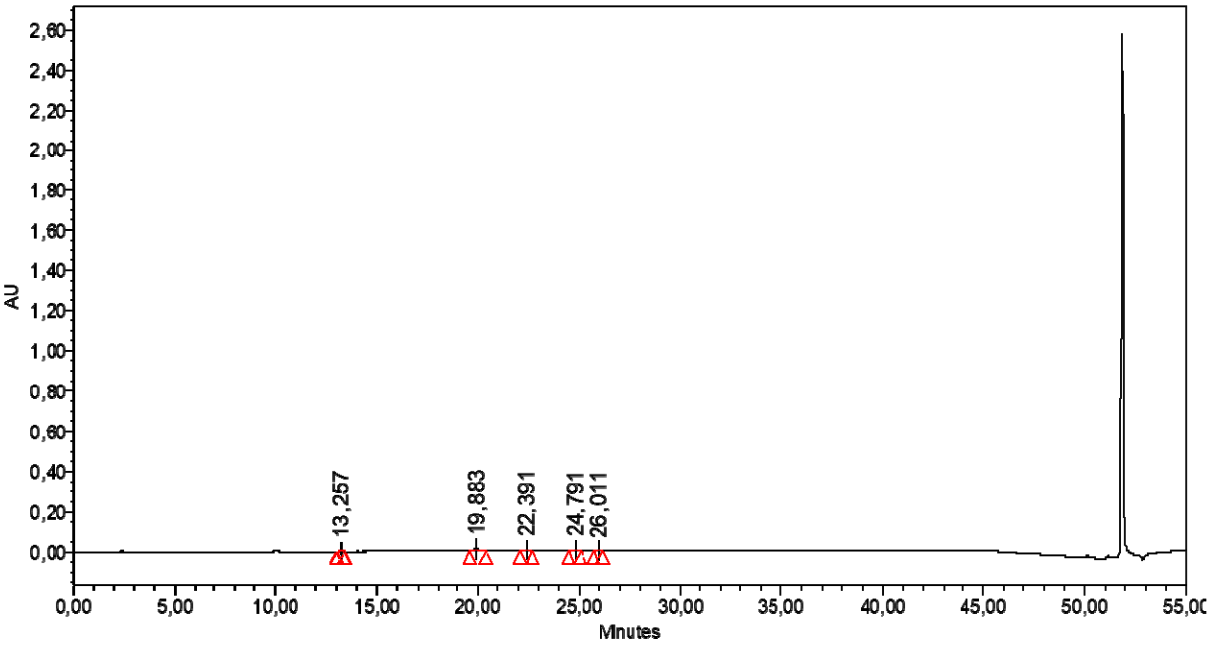

# Cannellino Bio
# 4CB extract

| p-OH benz | 3 OH benz | p-coumaric acid | Sinapinic acid | t-ferulic acid |
|---|---|---|---|---|
| 256 | 295 | 309 | 324 | 315 |

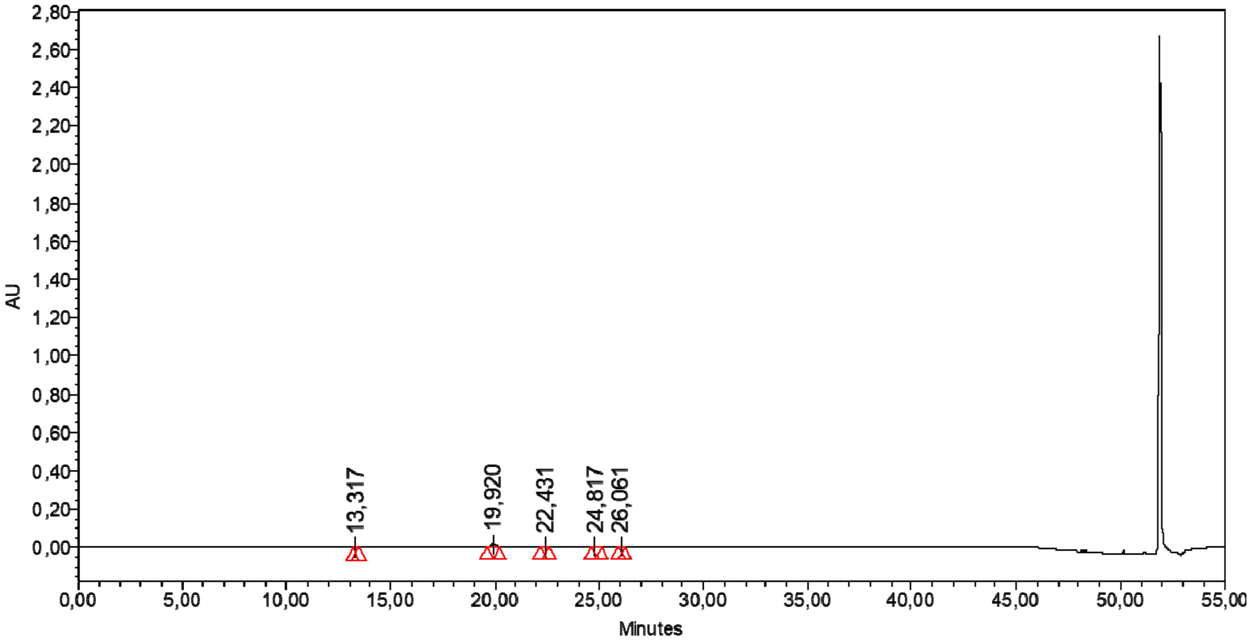


**HPLC traces of Cannellino Bio 4TT and 4CB extracts**
